# Biomimetic scaffold and 3D bioprinting in dental application: A review

**DOI:** 10.6026/973206300200789

**Published:** 2024-07-31

**Authors:** Uma Subbiah, Vijayalakshmi Rajaram, Jaideep Mahendra, Lakshmi Priya Kannan, Burnice Nalinakumari Chellathurai, Ambalavanan Namasivayam

**Affiliations:** 1 Department of Periodontics, Meenakshi Ammal Dental College and Hospital, Chennai, Tamil Nadu, India

**Keywords:** Tissue scaffolds, tissue engineering, regenerative medicine, three-dimensional printing

## Abstract

Biomimetic scaffold and 3D bioprinting technologies have emerged as promising avenues in tissue engineering and regenerative medicine, offering innovative approaches to address the limitations of conventional tissue engineering methods. This review article provides a comprehensive overview of recent advancements, challenges, and future prospects in the field of biomimetic scaffold fabrication and 3D bioprinting techniques.

## Background:

Periodontitis, an inflammatory disease, poses risks of tissue and bone loss, leading to compromised dentition. With the increasing prevalence of periodontitis, regenerative procedures are crucial to restore a healthy periodontium. [1, 2, 3-4] Periodontal therapy aims to regenerate the entire periodontal complex; Current nonsurgical and surgical techniques focus on tissue removal but often result in repair, lacking complete restoration. [5] Guided Tissue Regeneration (GTR) using biomaterials, considered the gold standard, seeks to inhibit epithelial cell down growth for selective repopulation of periodontal ligament and bone. [6] However, GTR has limitations and clinical variability. Tissue engineering introduces promising strategies by combining biomaterials, scaffolds, and stem cells. [6] Scaffolds provide contact guidance for cell migration, and bioactive molecules enhance tissue ingrowth. [7] Conventional techniques in tissue engineering fall short, prompting interest in individualized three-dimensional (3D) printed scaffolds. [2] This emerging technology allows precise control over macro- and micro-structure, replicating the complexity of periodontal tissue. Bioprinting, embedding living cells, further advances hierarchical architecture in 3D, offering potential applications in periodontal regeneration. [8] Therefore, it is of interest to report 3D bioprinting techniques, materials, and their applications, focusing on membranes and scaffolds for periodontal apparatus regeneration.

## Periodontal tissue engineering triad:

In 1993, Langer proposed tissue engineering as a method to regenerate lost periodontal tissues. [8] The technique aims to deliver biologically active elements that integrate into host tissues, resulting in three-dimensional regeneration similar to the lost tissues. The tissue engineering triad consists of cells, growth-stimulating signals, and scaffolds, where the scaffold serves as the cell niche, facilitating attachment, migration, proliferation, and 3D spatial organization. [9]

## Scaffold for periodontal regeneration:

The major functions of scaffolds include maintaining the shape of defects, offering physical support, serving as a 3D substratum for cellular activities, acting as a selective barrier for cellular migration, and potentially delivering growth factors. Biomimetic scaffolds, mimicking the extracellular matrix (ECM), have emerged as promising materials for tissue repair. [10] Advances in biomimetic scaffold engineering include prefabrication of 3D structures, surface modification to mimic biological environments, and the formation of in-situ gelled hydrogel scaffolds using biologically inspired materials through crosslinking processes. [11]

## Materials used for scaffold fabrication include:

Various materials are employed in the fabrication of scaffolds for tissue regeneration. One such material is decellularized extracellular matrix (ECM), sourced from human dermis, amniotic membrane, porcine collagen matrix, and small intestine submucosa. [12] Its advantages encompass the promotion of cellular migration, revascularization, and minimal patient morbidity. This material finds application in root coverage, augmenting tissue thickness, and serving as a standalone technology for periodontal and peri-implant soft tissue reconstruction. Organic materials including natural polymers like collagen, gelatin and chitosan exhibit biocompatibility with low mechanical strength. This makes them suitable for regenerating periodontal ligament, alveolar bone, and cementum [13, 14]. Synthetic polymers such as PLGA and PCL, with tunable degradation rates, are utilized for alveolar bone and periodontal ligament regeneration. Additionally, inorganic materials like hydroxyapatite (HA), tricalcium phosphate (TCP), biphasic calcium phosphate (BCP), and bioactive glass contribute to alveolar bone and cementum regeneration, offering properties such as osteoconductivity, direct bonding to natural bone, and diverse degradation rates. [15, 16]

## Scaffold fabrication techniques include:

Firstly, decellularization utilizes decellularized extracellular matrix (ECM) derived from human, porcine, or bovine dermis, as well as human amniotic membrane, to recreate a 3D microenvironment for tissue repair. [17, 18] This method, applied in clinical practice, reduces surgical time and morbidity compared to autogenous grafts. Salt leaching, commonly used in tissue engineering scaffolds, involves placing salt crystals in a mold, filling the spaces with polymer, and then dissolving the salt to achieve controlled pore size, though control over interpore openings and shape is limited. [19] Gas foaming utilizes gas as a porogen, eliminating harsh chemical solvents and reducing fabrication time, but challenges include ensuring pore connectivity and limited control over pore sizes. [20, 21, 22-23] Phase separation involves rapidly cooling a polymer dissolved in a solvent in a mold, with different techniques like thermally-induced, solid-liquid, and liquid-liquid phase separation available, although solvent use inhibits the incorporation of bioactive molecules or cells during scaffold fabrication. [24, 25] Lastly, freeze-drying, a conventional technique for 3D scaffold fabrication, involves sublimating freeze-dried materials to create pores, offering complex geometry and uniform pore morphology. Parameters like water-to-polymer ratio and emulsion viscosity influence scaffold porosity and pore sizes, with advantages including the elimination of rinsing steps, though control is necessary to reduce heterogeneous freezing for scaffold homogeneity. [25, 26, 23-28]

## Limitation of conventional 3D scaffolds:

The limitations of traditional scaffold fabrication techniques are evident in their lack of precise control over internal scaffold architecture and the creation of intricate structures. Achieving consistency in scaffold architecture demands proficient fabrication skills, posing a challenge for researchers and practitioners. Moreover, the reliance on harsh chemical solvents in these methods introduces a potential risk of cell death, as incomplete removal of toxic solvents may compromise the biocompatibility of the scaffold. Another drawback is observed in the compressive moduli of scaffolds produced through traditional techniques, which often fall significantly below the levels observed in hard or soft tissues. These constraints highlight the need for advancements in scaffold fabrication methods to address these issues and enhance the overall effectiveness of regenerative approaches in tissue engineering.

## 3D bioprinting:

Three-dimensional (3D) printing also called additive manufacturing is a process in which entities are fabricated by placing materials layer by layer to yield a three-dimensional assembly. This method can produce any 3D object with the help of computer aided design (CAD). [29, 30, 31- 32]

## Bioprinters:

Novel technology, known as bioprinting, facilitates the 3D printing of living cells and supporting components into intricate functional tissues. [33] Bioprinting enhances regenerative medicine by localizing various seed cells precisely, allowing for remarkable controllability over biomaterial positioning and maintaining accuracy in internal and external details. This technology is notably applied in dental regeneration, specifically in the regeneration of the periodontium. [7, 35]

## Widely used bioprinters:

Advanced bioprinting technologies offer innovative solutions in the field of tissue engineering. Inkjet Printing, a non-contact method, utilizes ink drops to reproduce data onto a substrate, overcoming initial challenges by encapsulating cells in hydrated hydrogel polymers. [33] Printer types, such as thermal, piezoelectric and mechanical, contribute to the versatility of this technique. Micro Extrusion Bioprinting employs fluid dispensing and robotic systems for structure extrusion, utilizing pneumatic, screw-driven, piston, or solenoid-based systems. [4c] Laser-Assisted Bioprinting (LAB) utilizes laser deposition of bio-ink on a substrate, offering a high-resolution scaffold-free technique that eliminates nozzle clogging. [4] However, drawbacks include the presence of metallic residues and high costs associated with this precise and efficient bioprinting method.

## Biological Inks (Bio-inks):

In the realm of bioprinting, diverse approaches contribute to the fabrication of intricate living tissues. Scaffold-based constructs utilize hydrogels extensively in Inkjet, laser-assisted, and extrusion-based bioprinting. While decellularized matrix-based bio-ink achieves good biomimicry, it may lack the mechanical strength required for constructing large-scale tissue. The incorporation of microcarriers enhances cellular attachments but introduces the risk of nozzle clogging. On the other hand, Scaffold-Free Bio-inks, applied in extrusion-based bioprinting, enable the creation of highly dense cellular constructs without the support of a hydrogel. This technique involves utilizing cell suspensions in growth media, and alternatives like tissue spheroids, cell pellets, and tissue strands serve as scaffold-free bio-inks. [36]

## Steps in bioprinting:

The 3D bioprinting process comprises three essential stages. Firstly, during the Pre-bioprinting phase, the 3D structure is designed and modeled using CT and MRI scans, with tomographic reconstruction capturing fine details for subsequent layer-by-layer printing. Bio-inks are prepared by isolating cells from living tissues and allowing them to multiply, setting the foundation for the subsequent printing process. Secondly, in the Bioprinting stage, the designed structures are brought to life using specialized printers. Bio-inks, containing cells, are introduced into printer cartridges, and the cells are precisely accumulated in a layered fashion, following the digital model. [8] Finally, in the Post-bioprinting phase, attention is focused on ensuring the mechanical integrity and functionality of the printed structure. This stage also involves controlling tissue remodeling and growth through signaling mechanisms. [9]

## Advantages and shortcomings of 3D bioprinting:

The advantages of 3D bioprinting are noteworthy, offering design flexibility with precise control over parameters such as porosity, pore size, interconnectivity, and strand alignment. [37] This capability is particularly advantageous for tailoring structures in periodontics and ensuring compatibility with diagnostic imaging equipment, enabling personalized medicine approaches. Additionally, 3D bioprinting contributes to improved predictability in periodontal therapy, especially for addressing advanced tissue defects. However, the cost factor is a significant concern, encompassing expensive 3D bioprinters, high energy consumption, and ongoing operation and maintenance expenses. [38] Furthermore, the requirement for trained operators acts as a potential barrier for the widespread development and adoption of 3D bioprinting technologies in various medical applications.

## Application in periodontics:

Polycaprolactone (PCL) Fused deposition modeling (FDM) specifically designed plugs are used for alveolar ridge preservation with some success providing an alternative to particulate synthetic calcium phosphate or deproteinized xenograft materials. A customized scaffold was 3D printed using medical grade polycaprolactone to fit the periosseous defect using a prototype model of the defect from the patient's CBCT. Scaffold matrix was placed onto the defect and post-operative follow-up was done. 3D bioprinting can be used to create customized scaffolds that mimic the complex architecture of periodontal tissues such as gingiva, periodontal ligament, and alveolar bone. These scaffolds can serve as a framework for tissue regeneration in patients with periodontal disease or trauma. [39] By precisely depositing biocompatible materials and cells layer by layer, 3D bioprinting enables the fabrication of periodontal tissue constructs with controlled microarchitecture and allows for the replacement of damaged or lost periodontal tissues. [40] 3D bioprinting can be utilized to create drug delivery systems tailored for periodontal applications and can be designed to release therapeutic agents such as antibiotics, growth factors, or anti-inflammatory drugs directly into the periodontal pocket, enhancing treatment efficacy and reducing side effects. With advances in imaging technology such as cone-beam computed tomography (CBCT) and intraoral scanning, patient-specific anatomical data can be obtained. By integrating this data with 3D bioprinting technology, clinicians can create personalized treatment plans and fabricate patient-specific grafts or implants for periodontal defect repair. [41] 3D bioprinting can be used to create anatomically accurate models of periodontal structures for educational and training purposes. [42] 3D bioprinting provides researchers with a powerful tool to study periodontal diseases, and tissue regeneration mechanisms. This technology facilitates the development of new therapeutic approaches and the evaluation of their potential clinical applications in periodontology. [42] Overall, 3D bioprinting has the potential to revolutionize various aspects of periodontal care, ranging from tissue regeneration to personalized treatment planning, thereby improving patient outcomes and advancing the field of periodontology.

## Conclusion:

3D bio-printing has found practical use in periodontics, demonstrating promising outcomes in alveolar ridge preservation and peri osseous defect management. Ongoing research will be pivotal in addressing limitations and refining the technology, unlocking the full potential of 3D bioprinting to revolutionize periodontal regeneration. This era in periodontology signifies an exciting intersection of technology and biological sciences, paving the way for precision, personalization, and improved patient outcomes.
